# Integrated AAV optimization enables efficient gene delivery to kidney in murine and human tissue

**DOI:** 10.21203/rs.3.rs-7925411/v1

**Published:** 2025-10-24

**Authors:** Fernando Gomez Garcia, Miriam Rivera Moreno, Shashank Chetty, Najani Johnson, Andrea An, Juliet Sostena, Lily Thompson, Jing Wang, Vivek Charu, Jean Lee, Natalie Meyer, Marshall Stoller, Heiko Yang, Nabil Boutagy, Jenna DiRito, Greg Tietjen, Catarina Allegue Toscano, Miguel Garcia-Gonzalez, George Church, Mark Kay, Avnesh Thakor, Vivek Bhalla, Demetrios Maxim

**Affiliations:** Nephrogen; Nephrogen; Stanford University; Nephrogen; Nephrogen; Nephrogen; Nephrogen; Stanford University; Stanford University; University of Colorado Anschutz School of Medicine; University of Colorado Anschutz School of Medicine; University of California, San Francisco; University of California, San Francisco; Revalia Bio; Revalia Bio; Revalia Bio; Center for Research in Molecular Medicine and Chronic Diseases (CiMUS); Instituto de Investigación Sanitaria; Harvard Medical School; Stanford University; Stanford University; Stanford University School of Medicine; Nephrogen

## Abstract

Unbiased screening of patients seen in nephrology clinics indicate that ~ 10% of patients have a monogenetic cause of kidney disease. For these patients and possibly for the broader diaspora of patients living with chronic kidney disease, gene therapy may be efficacious and possibly curative. A major limitation of gene therapy to the kidney parenchyma thus far, has been seemingly poor delivery of cargo to cell types of interest, utilizing existing adeno-associated viral capsids (e.g. AAV9). Novel AAV capsid serotypes may enhance transduction efficiency, but compared to FDA-approved capsids for extrarenal diseases, safety and tolerability data in humans is unknown. We systematically varied promoter, cargo, genome configuration, enzymatic priming, capsid serotype, dose, and route of administration, and show that optimizing these parameters yields some of the highest kidney transduction efficiencies reported in mice—40 to 60% of kidney tubules transduced with systemic AAV9-Cbh-mCherry-WPRE—by multiple unbiased quantification methods, including a novel machine-learning-based image analysis tool. We also demonstrate AAV transduction in live human kidney using *ex vivo* normothermic perfusion. These data provide a roadmap for ongoing preclinical studies to enable translation of AAV-mediated kidney gene therapy into the clinic.

## INTRODUCTION

Chronic kidney disease represents a major global health burden, affecting ~ 8–16% of the population and showing a sharp rise in incidence over the last decade.^1^ At least 10% of patients with chronic kidney disease have monogenetic kidney disease,^2^ including conditions such as polycystic kidney disease, type IV collagenopathies, Dent disease and cystinosis.^2,3^ These inherited disorders lack curative treatments, and disease progression often culminates in end stage kidney disease, for which only dialysis or kidney transplantation remain the primary therapeutic options. While dialysis is associated with high morbidity and reduced quality of life, transplantation is constrained by scarcity of donor organs and the requirement for lifelong immunosuppression.^4^ These challenges highlight the urgent need for innovative approaches that can target the underlying genetic causes of kidney disease. In this context, gene therapy has emerged as a promising strategy capable of providing long-term therapeutic benefit through precise and durable genetic correction within kidney tissue.

Recombinant adeno-associated virus (AAV) vectors have emerged as powerful vehicles for *in vivo* gene delivery due to their favorable safety profile, long-term expression capacity, and broad tissue tropism. AAV-mediated delivery of genome editing tools has shown remarkable efficiency in various tissues including the brain, liver, retina, and skeletal muscle.^5–7^ In these organs, AAV-based strategies have not only enabled efficient gene transfer but also led to functional improvements in preclinical and clinical settings. Although the application of genome editing technologies to treat kidney diseases lags behind other tissues, these tools have been used extensively to generate disease models that advance our understanding of renal pathophysiology.^8,9^ Notably, recent studies have achieved AAV-mediated delivery of therapeutic genes to glomerular and cystic cell types, resulting in measurable phenotypic improvements in experimental models of nephrotic syndrome, glomerulonephritis and autosomal dominant polycystic kidney disease.^10–12^ Despite these advances, the determinants of efficient AAV-mediated gene delivery to the kidney are not well known, particularly with regard to targeting of proximal tubular epithelial cells—the principal site of injury in many inherited nephropathies.^13,14^ While most early approaches showed limited efficacy in this kidney compartment, recent strategies incorporating engineered AAV capsids and localized delivery routes have begun to demonstrate promising levels of transgene expression in tubular compartments.^15,16^

In this study, we present a systematic and comprehensive evaluation of key AAV vector components—promoter, cargo, genome configuration, priming by enzymatic digestion, capsid serotype, dose, and delivery route—with the aim of optimizing kidney gene transfer, particularly to proximal tubular cells. Furthermore, we validate the translational potential in an *ex vivo* human kidney perfusion model. Our results provide a practical roadmap for the development of AAV-based gene therapies and establish a foundational platform for advanced gene therapy technologies to treat kidney disease.

## RESULTS

### CBH and CAG promoters drive robust reporter gene expression in mouse kidney tubular epithelial cells following systemic AAV9 administration

**Figure 1:**
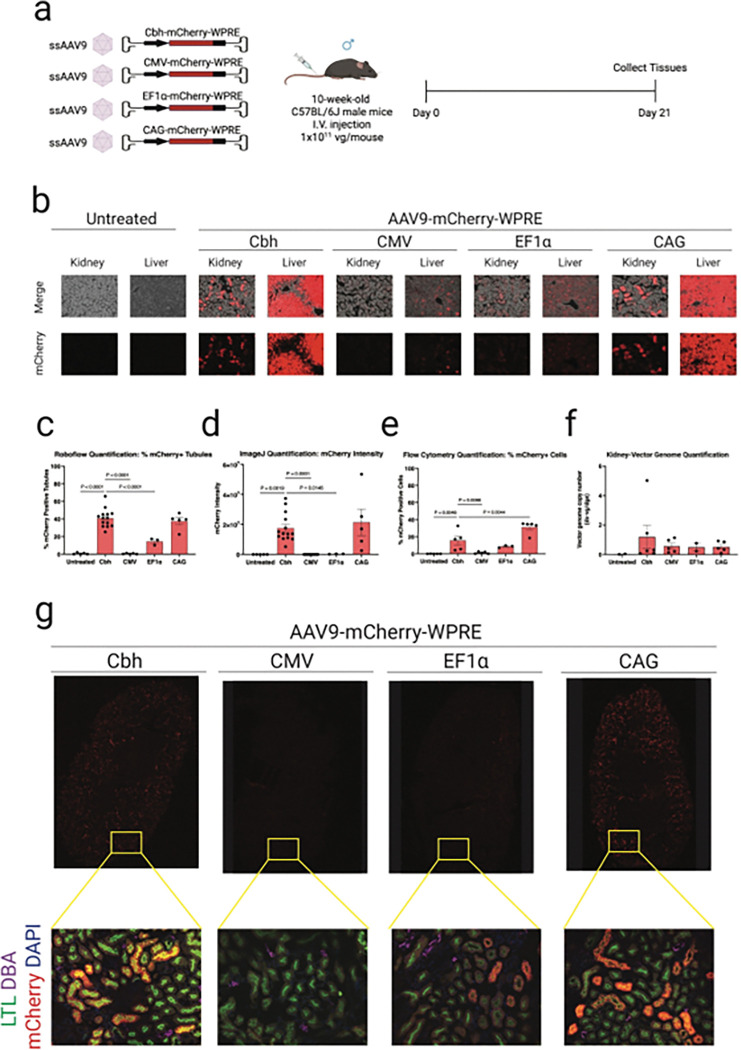
Promoter-dependent AAV9-mediated gene expression in mouse kidney. **a**, Experimental design. Ten-week-old male C57BL/6J mice were injected intravenously with saline solution, AAV9-CBH-mCherry, AAV9-CMV-mCherry, AAV9-EF1α-mCherry or AAV9-CAG-mCherry at a dose of 1.0 × 10^11^ vg/mouse (N = 5, 12, 5, 3 and 5 per group, respectively). Kidneys and livers were harvested three weeks post-injection for downstream analyses. **b**, Representative images of mCherry fluorescence in the kidney and liver of AAV-treated vs. untreated mice. **c**, Transduction efficiencies in AAV-treated vs. untreated renal tubules were quantified using Roboflow machine learning-guided image analysis tool. **d**, mCherry fluorescence intensity in AAV-treated vs. untreated renal tubules was quantified using ImageJ. **e**, mCherry transduction efficiency in kidney cells from AAV-treated vs untreated mice was quantified by flow cytometry. **f**, Representative confocal microscopic images of the kidney from AAV-treated mice. Red, mCherry fluorescence; blue, DAPI; green, LTL; purple, DBA. Data are presented as mean ± s.e.m. Statistical analysis was performed using one-way ANOVA. Only statistically significant *P* values are reported. *P* < 0.05 was considered statistically significant. Source data and exact *P* values are provided in the Source Data file.

To investigate how promoter selection influences kidney transgene expression, we engineered AAV9 vectors encoding the fluorescent reporter mCherry under the control of four promoters: chicken beta-actin hybrid (CBH), cytomegalovirus (CMV), elongation factor 1 alpha (EF1α), and CAG. Kidneys and livers were collected 21 days post-injection for comparative analysis (Fig. 1a). Representative fluorescence images of kidney and liver tissue revealed striking differences in transgene expression driven by these various promoters (Fig. 1b). CBH and CAG promoters induced mCherry expression in 41% ± 3% and 38% ± 4% of kidney tubules, respectively, which was significantly greater than the percentage of mCherry expressing tubules in mice treated with the CMV and EF1α promoters (0.5% ± 0.2% and 15% ± 2%, respectively). Image quantification of mCherry-positive kidney tubules using an unbiased machine learning model (Supplementary Fig. 1) confirmed this trend, with CBH and CAG promoters activating expression in significantly more tubules than CMV by 90-fold and 82-fold respectively (both P < 0.0001). CBH and CAG promoters also stimulated expression in significantly more tubules than EF1α by 3-fold (P < 0.0001) and 3-fold (P = 0.0019), respectively (Fig. 1c). Consistent with tubule counts, per-tubule mCherry fluorescence intensity was markedly higher with CBH and CAG than with CMV or EF1α. Relative to CMV, CBH and CAG increased intensity by 5,862-fold (P = 0.0100) and 7,161-fold (P = 0.0086), respectively; and relative to EF1α, increased intensity by 64-fold (P = 0.0465) and 78-fold (P = 0.0272), respectively (Fig. 1d). Flow cytometry further corroborated differences in expression at the single-cell level. Relative to CMV, CBH and CAG stimulated expression in significantly more cells than CMV by 13-fold (P = 0.0086) and 26-fold (P < 0.0001), respectively and relative to EF1α by 2-fold (not significant) and 4-fold (P = 0.0005), respectively (Fig. 1e; Supplementary Fig. 2). We show that these two unbiased methods for quantifying kidney transduction, machine-learning-based quantitative immunofluorescence and flow cytometry, are directly correlated in individual mice (P = 0.0034, Supplementary Fig. 1c). Despite the stronger mCherry signal observed in kidney with CBH and CAG promoters, vector genomes were not significantly higher in mice injected with CBH and CAG vs. EF1α or CMV (Fig. 1f). These data suggest that expression, not tropism, is the primary barrier to AAV9-mediated kidney gene transfer.

Next, we sought to determine the nephron segment specificity of AAV9 using lectin-based markers of proximal and distal tubular cells (Fig. 1f). mCherry expression driven by either the CBH or CAG promoter localized predominantly to proximal tubules within kidney cortex as indicated by positive staining for lotus tetragonolobus lectin (LTL), with minimal to no overlap observed with markers for collecting duct, suggesting that AAV9 favors transduction of kidney proximal tubular epithelial cells. To confirm this observation, we performed flow cytometry of kidney single-cell suspensions using LTL and mCherry labeling (Supplementary Fig. 3). In mice injected with AAV9-CBH-mCherry, we found that ~ 60% of mCherry-positive cells were LTL-positive via flow cytometry (Supplementary Fig. 3), which suggests that AAV9-CBH primarily expresses in LTL + proximal tubular cells. Qualitatively, mCherry expression with AAV9-CBH was not observed in glomerular cells nor in DBA + principal cells of the collecting duct (Fig. 1g), suggesting that the remainder of kidney cell types where AAV9-CBH expresses are LTL-negative proximal tubular cells, cells from the Loop of Henle, or possibly distal convoluted tubule.

To compare AAV9 with non-viral delivery strategies, we tested three lipid nanoparticle (LNP) formulations used in clinically approved drugs—ALC-0315, SM-102, and DLin-MC3—each packaged with an mCherry-encoding mRNA. All three LNPs transduced > 98% of kidney cells *in vitro* (Supplementary Fig. 4a). However, none of these LNPs achieved detectable mCherry expression in kidney when administered intravenously (Supplementary Fig. 4b–d).

Taken together, these results demonstrate that systemic delivery of AAV9 vectors using either the CBH or CAG promoter results in robust and preferential transgene expression in proximal tubular epithelia. Given the therapeutic relevance of targeting this nephron segment in genetic kidney diseases, these findings establish both promoters as strong candidates for future gene therapy strategies. Despite their comparable efficacy, we selected the CBH promoter for subsequent experiments since it is ~ 50% shorter than the CAG promoter (793 bp vs. 1,701 bp, respectively) (Supplementary Fig. 5a) and hence would be better suited for more efficient viral packaging of larger cargo.

### Transgene identity affects kidney expression independent of the viral capsid and expression cassette

**Figure 2:**
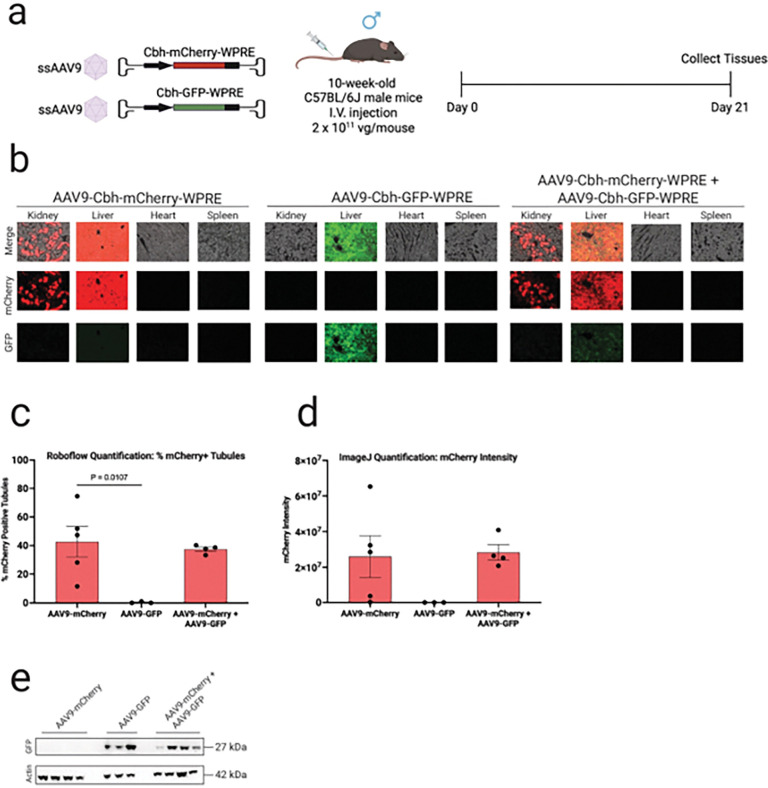
mCherry detection in mouse kidney is more robust than GFP. **a**, Experimental design. Ten-week-old male C57BL/6J mice were injected intravenously with AAV9-CBH-mCherry, AAV9-CBH-GFP or both at a dose of 2.0 × 10^11^ vg/mouse (N = 5, 2, and 4 per group, respectively). Kidneys, livers, hearts and spleens were harvested three weeks post-injection for downstream analyses. **b**, Representative images of mCherry and GFP fluorescence in the kidney, liver heart and spleen of AAV-treated mice. **c**, Transduction efficiencies in AAV-treated renal tubules were quantified using Roboflow machine learning-guided image analysis tool. **d**, mCherry fluorescence intensity in AAV-treated renal tubules was quantified using ImageJ. **e**, mCherry transduction efficiency in kidney cells from AAV-treated mice was quantified by flow cytometry. Red, mCherry fluorescence; green, GFP fluorescence. Data are presented as mean ± s.e.m. Statistical analysis was performed using one-way ANOVA. Only statistically significant *P* values are reported. *P*< 0.05 was considered statistically significant. Source data and exact *P*values are provided in the Source Data file.

To assess whether the transgene payload influences kidney expression, we used AAV9 vectors encoding either mCherry and/or GFP under the control of the CBH promoter. Representative fluorescence images of kidney, liver, heart, and spleen revealed marked differences in tissue-specific expression between the two transgenes (Fig. 2b). mCherry expression was detectable via immunofluorescence in both kidneys and livers of mice treated with AAV9-CBH-mCherry, but no GFP expression was detectable via immunofluorescence for AAV9-CBH-GFP in kidney. These findings were further supported in co-injected mice, whereby mCherry—but not GFP—fluorescence was observed in kidney, despite comparable expression of both fluorophores in the liver.

Quantification of mCherry-positive tubules and corresponding fluorescence intensity confirmed robust kidney transduction in the AAV9-CBH-mCherry group, as well as in co-injected mice (Fig. 2c–d), while no GFP signal was detected in kidneys of mice injected with AAV9-CBH-GFP alone. On the other hand, by immunoblotting, GFP protein was detected in kidneys of mice injected with AAV9-CBH-GFP, as well as in mice co-injected with AAV9-CBH-GFP and AAV9-CBH-mCherry, confirming that AAV-CBH-GFP transduces and expresses in kidney cells (Fig. 2e). These findings indicate that the lack of GFP fluorescence by conventional microscopy may not indicate lack of expression, but rather potential detection limitations. We selected mCherry as the preferred reporter for quantitative immunofluorescence for subsequent experiments.

### Self-complementary AAV9 vectors enhance kidney transgene expression intensity without increasing the number of tubules transduced

**Figure 3:**
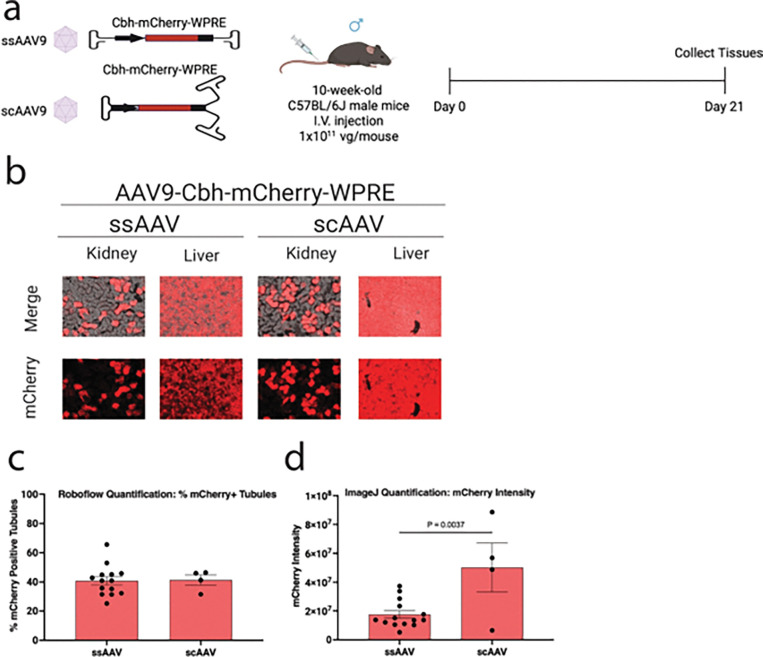
Self-complementary AAV9 shows comparable *in vivo* transduction efficiency but greater expression intensity than single-stranded AAV9 in mouse kidney. **a**, Experimental design. Ten-week-old male C57BL/6J mice were injected intravenously with ssAAV9-CBH-mCherry or scAAV9-CBH-mCherry at a dose of 1.0 × 10^11^ vg/mouse (N = 12 and 4 per group, respectively). Kidneys and liver were harvested three weeks post-injection for downstream analyses. **b**, Representative images of mCherry fluorescence in the kidney and liver of AAV-treated mice. **c**, Transduction efficiencies in AAV-treated renal tubules were quantified using Roboflow machine learning-guided image analysis tool. **d**, mCherry fluorescence intensity in AAV-treated renal tubules was quantified using ImageJ. Red, mCherry fluorescence. Data are presented as mean ± s.e.m. Statistical analysis was performed using unpaired t test. Only statistically significant *P*values are reported. *P* < 0.05 was considered statistically significant. Source data and exact *P* values are provided in the Source Data file.

To assess whether the configuration of the AAV genome affects kidney transgene expression, we compared single-stranded AAV9 (ssAAV9) and self-complementary AAV9 (scAAV9) vectors, both carrying the CBH-mCherry-WPRE cassette. Representative fluorescence images revealed strong mCherry expression in both the kidney and liver across groups (Fig. 3b). Quantification of mCherry-positive tubules demonstrated comparable overall kidney transduction between ssAAV9- and scAAV9-treated mice (40.8% ± 2.8% vs. 41.4% ± 3.5%, Fig. 3c), indicating that the number of transduced tubules was not affected by genome configuration (P = 0.9203). In contrast, analysis of mCherry fluorescence intensity revealed significantly stronger signal within individual tubules in the scAAV9 group, with a 2.86-fold increase in intensity compared to ssAAV9 (P = 0.0037) (Fig. 3c).

### Neuraminidase treatment does not enhance AAV9-mediated transduction of tubules

**Figure 4:**
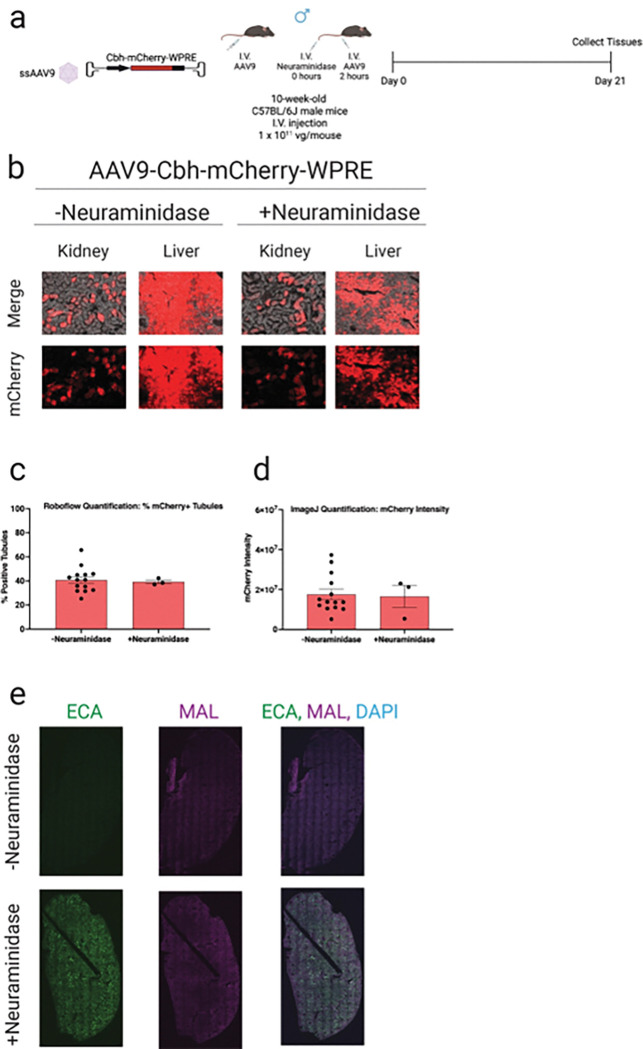
Neuraminidase priming does not significantly improve AAV9 kidney tropism in mice. **a**, Experimental design. Ten-week-old male C57BL/6J mice were injected intravenously with AAV9-CBH-mCherry or AAV9-CBH-mCherry plus Neuraminidase (200 milliunits, 2 hours before AAV injection) at a dose of 1.0 × 10^11^ vg/mouse (N = 12 and 3 per group, respectively). Kidneys and liver were harvested 3 three weeks post-injection for downstream analyses. **b**, Representative images of mCherry fluorescence in the kidney and liver of AAV-treated mice. **c**, Transduction efficiencies in AAV-renal tubules were quantified using Roboflow machine learning-guided image analysis tool. **d**, mCherry fluorescence intensity in AAV-treated renal tubules was quantified using ImageJ. **e,** Representative confocal microscopic images of the kidney from AAV-treated mice. Green, ECA; purple, MAL; blue, DAPI. Data are presented as mean ± s.e.m. Statistical analysis was performed using unpaired t test. Only statistically significant *P* values are reported. *P*< 0.05 was considered statistically significant. Source data and exact *P*values are provided in the Source Data file.

To explore whether modulating cell surface glycans could enhance AAV9-mediated transduction via increased tropism to the kidney, we tested the effect of neuraminidase pretreatment on transgene delivery (Fig. 4a). Neuraminidase cleaves terminal sialic acid residues from glycoproteins and glycolipids, thereby exposing underlying sugar moieties such as galactose and N-acetylgalactosamine, which are recognized by various AAV serotypes.^17^ Given that AAV9 uses terminal galactose as a primary receptor, we hypothesized that removing sialic acid to expose the terminal galactose residues would improve vector binding and uptake in kidney cells.

We utilized *Erythrina cristagalli agglutinin* (ECA) lectin, which binds β1,4-linked galactose, and *Maackia amurensis lectin II* (MAL-II), which binds α2,3-linked sialic acids, to confirm the change in receptor expression that we anticipated following neuraminidase administration. Kidneys from neuraminidase-treated mice displayed a marked increase in *Erythrina cristagalli agglutinin* expression compared to controls, confirming effective removal of sialic acid residues (Fig. 4e). In contrast, *Maackia amurensis lectin II* staining remained comparable between groups, likely reflecting incomplete desialylation or selective removal of specific sialyl linkages. Quantification following AAV9-Cbh-mCherry injection in mice pre-treated with neuraminidase vs. saline pre-treated controls showed no significant increases in number of mCherry-positive tubules nor signal intensity of mCherry-positive tubules between untreated and neuraminidase-pretreated mice (Fig. 4b–d), indicating that desialylation did not enhance AAV9-mediated kidney transduction under these conditions.

#### Capsid selection and dose escalation reveal a plateau in targetability but not in intensity of transgene expression

**Figure 5:**
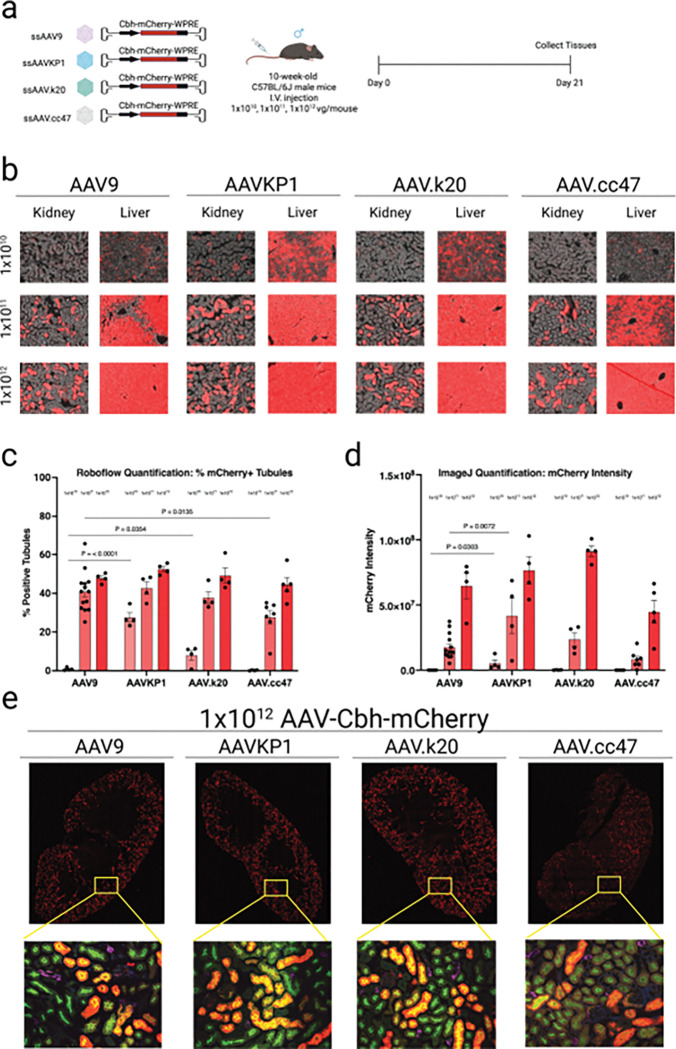
AAV9 shows comparable kidney transduction to novel engineered capsids using the CBH promoter. **a**, Experimental design. Ten-week-old male C57BL/6J mice were injected intravenously with AAV9-CBH-mCherry, AAVKP1-CBH-mCherry, AAV.k20-CBH-mCherry, or AAV.cc47-CBH-mCherry at a dose of 1.0 × 10^10^, 1.0 × 10^11^ or 1.0 × 10^12^ vg/mouse (N: AAV9= 4, 12 and 3;; AAVKP1= 4, 4 and 4; AAV.k20 = 4, 4 and 4; AAV.cc47= 4, 7 and 5 per group and per dosage, respectively). Kidneys and livers were harvested three weeks post-injection for downstream analyses. **b**, Representative images of mCherry fluorescence in the kidney and liver of AAV-treated mice. **c**, Transduction efficiencies in AAV-treated renal tubules were quantified using Roboflow machine learning-guided image analysis tool. **d**, mCherry fluorescence intensity in AAV-treated renal tubules was quantified using ImageJ. **e**, Representative confocal microscopic images of kidneys from mice treated with 1.0 × 10^12^ vg. Red, mCherry fluorescence; blue, DAPI; green, LTL; purple, DBA. Data are presented as mean ± s.e.m. Statistical analysis was performed using one-way ANOVA. Only statistically significant *P* values are reported. *P* < 0.05 was considered statistically significant. Source data and exact *P* values are provided in the Source Data file.

To evaluate how AAV capsid selection and vector dose influence kidney gene transfer, we compared a conventional serotype (AAV9) with three engineered capsids (AAV-KP1, AAV.k20, AAV.cc47), each carrying the CBH-mCherry cassette, across escalating intravenous doses (1×10^10^, 1×10^11^, and 1×10^12^ vg per mouse) (Fig. 5a). AAV-KP1 is a novel AAV capsid developed by our team through AAV shuffling^18^ and is known to transduce kidney tubular epithelial cells in mice and non-human primates.^19^ Both AAV.k20 and AAV.cc47 are modified AAV capsids developed by Aravind Asokan and colleagues via 7-mer peptide insertion in AAV9 variable region IV and were previously shown to transduce kidney tubular epithelial cells in mice.^16,20^ Representative fluorescence images of kidney and liver sections revealed that all four synthetic capsids supported dose-dependent expression in both kidney and liver (Fig. 5b).

At 1×10^10^ vg, AAV-KP1 and AAV.k20 yielded a higher fraction of mCherry positive tubules than AAV9 (AAV-KP1 vs AAV9, P < 0.001; AAV.k20 vs AAV9, P = 0.0354). At 1×10^11^ vg, AAV9 outperformed AAV.cc47 (P = 0.0135). At 1×10^12^ vg, no inter-capsid differences were detected. Quantification of mCherry intensity (Fig. 5d) showed a robust dose–response for all capsids, with signal increasing from 1×10^10^ to 1×10^11^ to 1×10^12^ vg. Inter-capsid differences were limited to the lower doses—AAV-KP1 > AAV9 at 1×10^10^ (P = 0.0303) and 1×10^11^ (P = 0.0072). At 1×10^12^ vg, mCherry intensities were indistinguishable across capsids, indicating that dose escalation yields comparable expression strength among vectors.

Confocal imaging of whole kidney sections stained with nephron segment-specific lectins, showed that all four synthetic capsids primarily transduced proximal tubules, as evidenced by colocalization of mCherry with LTL-positive tubules and absence of overlap with DBA-positive tubules (Fig. 5f).

Collectively, these findings indicate that AAV-KP1, AAV.k20, and AAV.cc47 transduce kidney with similar efficiencies as AAV9 using the AAV-CBH-mCherry transgene cassette. While capsid choice did not significantly affect targeting within the tested dose range, vector dose played a critical role in intensity of expression.

#### Local renal arterial injection does not enhance AAV transduction efficiency in kidney tubules compared to systemic intravenous delivery in mice

**Figure 6:**
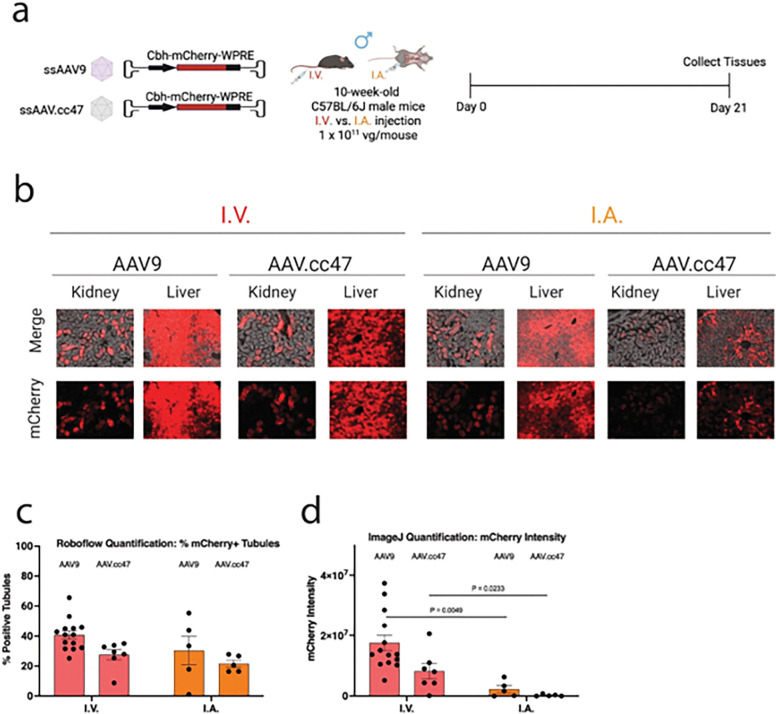
Systemic intravenous and local renal-artery injection show comparable kidney transduction with AAV9 and AAV.cc47 in mice. **a**, Experimental design. Ten-week-old male C57BL/6J mice were injected with AAV9-CBH-mCherry or AAV.cc47-CBH-mCherry via AAV9-CBH-mCherry via intravenous or renal artery at a dose of 1.0 × 10^11^ vg/mouse (N: AAV9= 12 and 5; AAV.cc47= 7 and 5, per group, respectively). Kidneys and liver were harvested three weeks post-injection for downstream analyses. **b**, Representative images of mCherry fluorescence in the kidney and liver of AAV-treated mice. **c**, Transduction efficiencies in AAV-treated renal tubules were quantified using Roboflow machine learning-guided image analysis tool. **d**, mCherry fluorescence intensity in AAV-treated renal tubules was quantified using ImageJ. Red, mCherry fluorescence using AAV9; orange, mCherry fluorescence using AAV.cc47 Data are presented as mean ± s.e.m. Statistical analysis was performed using unpaired t test. Only statistically significant *P*values are reported. *P* < 0.05 was considered statistically significant. Source data and exact *P* values are provided in the Source Data file.

To evaluate whether local kidney delivery enhances transduction efficiency, we compared systemic (intravenous, I.V.) versus local (intra-renal artery, I.A.) administration of AAV9 and AAV.cc47, both encoding the CBH-mCherry-WPRE cassette (Fig. 6a). In humans, AAV can be dosed via catheterization of the renal artery; however, the renal artery is too small to catheterize in mice. Therefore, we employed a surgical approach, using a series of temporary ligations, to replicate intra-renal administration in mice and confirmed that our approach is effective for reaching kidney cells by injecting a Trypan blue visualization dye (Supplementary Fig. 6).^21^

Representative fluorescence images of kidney and liver revealed that I.V. delivery through the systemic circulation led to stronger and more widespread mCherry expression in the kidney compared to I.A. injection for both AAV9 and AAV.cc47 (Fig. 6b). Notably, liver expression was markedly reduced following I.A. administration, indicating successful restriction of vector dissemination to the systemic circulation. The percentage of mCherry-positive tubules was not statistically different between I.V. vs I.A. injection for neither AAV9 (40.8% ± 2.8% vs. 30.3% ± 9.6%, respectively) nor AAV.cc47 (27.6% ± 3.4% vs. 21.6% ± 2.5%, respectively), suggesting that local I.A. and systemic I.V. injection routes transduce a similar fraction of tubules (Fig. 6c). However, mCherry fluorescence intensity was significantly higher in the I.V. group for both AAV9 (*P* = 0.0049) and AAV.cc47 (*P* = 0.0233) (Fig. 6d), indicating more efficient transgene expression at the cellular level following systemic delivery. These findings highlight that route of administration can impact not only the distribution of transduction but also the magnitude of gene expression within target cells, even when the number of transduced tubules remains comparable.

### AAV9 transduces kidney tubular epithelial cells in live human kidney following ex vivo perfusion

**Figure 7:**
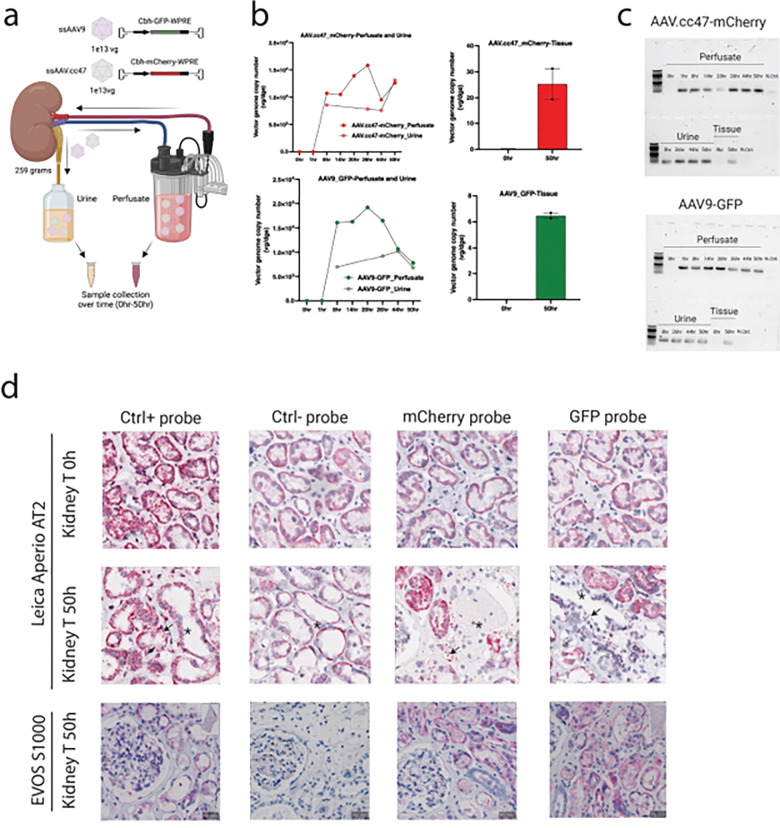
AAV9 and AAV.cc47 display kidney tropism in live human kidney via normothermic ex vivo perfusion. **a,** Experimental design. A male donor kidney was perfused with a mixed solution containing 1×10^13^ vg each of ssAAV9-CBH-GFP-WPRE and ssAAV.cc47-CBH-mCherry-WPRE for 50 hours. Vector was added 2 hours after starting perfusion. Tissue, urine and perfusate were collected at multiple time points for downstream analysis. **b**, Vector genome copy number was measured over the time in samples of perfusate, urine and tissue by qPCR and expressed as fold change relative to the initial time point (0 h). Line graphs show temporal dynamics of ssAAV.cc47-CBH-mCherry (top) and AAV9-CBH-GFP (bottom) in perfusate and urine. Bar graphs represent AAV vector genome copy number in tissue samples collected at the beginning and end of perfusion. **c**, Agarose gel electrophoresis of PCR amplicons generated from DNA extracted from perfusate, urine, and tissue samples collected at multiple time points during ex vivo perfusion experiments. Primers specific for mCherry (top panel) and GFP (bottom panel) were used to detect the presence of vector genomes. The gels show the distribution and persistence of each vector in fluid and tissue compartments throughout the duration of the experiments. Each lane represents a distinct time point or tissue sample, as indicated by the labels above the gels. **d,** Representative microscope images of human kidney tissue analyzed by RNAscope^®^ 2.5 assay using probes targeting the anti-sense strand for detection of vector genomes. In situ hybridization was performed on sections collected at the start (T0h) and end (T50h) of the ex vivo perfusion. Red dots (arrows) indicate vector-derived DNA transcripts of mCherry or GFP, confirming kidney transduction. Blue dots (asterisks) represent DAPI-stained nuclei.

To evaluate the translational potential of AAV9 and AAV.cc47, we utilized normothermic kidney perfusion to prolong survival and function of human kidneys *ex vivo*. A single kidney was perfused with a mixture of AAV.cc47-CBH-mCherry-WPRE and AAV9-CBH-GFP-WPRE simultaneously at a dose of 1 × 10^13^ vg per virus. Urine/perfusate samples and tissue biopsies were collected over time for analysis (Fig. 7a). To quantify vector dynamics during the perfusion, we measured vector genome copies per diploid genome equivalent (vg/dge) in perfusate, urine, and tissue samples collected over time (Fig. 7b). First, we confirmed that our system was negative for pre-existing anti-AAV9 antibodies by screening the serum and perfusate samples with an anti-AAV9 total antibody ELISA (Supplementary Fig. 7). For both AAV.cc47-mCherry and AAV9-GFP vectors, we observed a progressive decline in vg/dge levels in the perfusate, indicating vector clearance from circulation. Notably, this clearance was only partially mirrored by an increase in vector genomes detected in the urine, suggesting that urinary excretion accounts for only a fraction of the viral load. Using endpoint PCR performed on DNA extracted from perfusate, urine, and tissue at various time points (Fig. 7c), we revealed a strong and sustained presence of viral genomes in the kidney and a progressive clearance from the circulation.

To substantiate these findings and assess kidney compartment specificity for AAV9 and AAV.cc47 transduction, we performed RNAscope^®^ in situ hybridization on kidney sections collected at baseline (T0h) and after 50 hours of perfusion (T50h) (Fig. 7d). At T0h, we observed signal from the positive control probe (human PPIB-cyclophilin B) but not from DNA probes targeting GFP or mCherry, as expected in the absence of viral exposure. In contrast, tissue at T50h displayed strong and specific signal with GFP and mCherry probes, which suggest efficient transduction by AAV9 and AAV.cc47, respectively. Upon visual inspection, the positive signal for both mCherry and GFP probes is visible in tubular fractions (Fig. 7d). The negative control probe did not generate a positive signal, validating probe specificity and assay performance. Collectively, these results establish the feasibility of efficient AAV delivery and transgene expression in human kidney tissue using an *ex vivo* perfusion model and corroborate our findings in mice that AAV9 may be a translationally relevant delivery vehicle for kidney gene therapy.

## DISCUSSION

In this study, we present a comprehensive evaluation of key parameters for AAV-mediated gene therapy to the kidney. By integrating vector design (promoter, cargo, genome configuration, capsid serotype, and dose) and delivery (prophylactic enzyme therapy, route of administration) characteristics, we establish a detailed framework for enhancing kidney gene transfer.

A major strength of our approach was the quantification of efficiency by distinct methods: 1) quantitative immunofluorescence using a machine learning model, 2) quantitative immunofluorescence for expression intensity, and 3) flow cytometry. We demonstrate that robust gene expression in the kidney can be achieved using AAV9—a serotype with robust safety and tolerability data in humans,^22^ traditionally considered suboptimal for kidney targeting. Our findings show that, when combined with rational expression cassette design incorporating potent yet compact regulatory elements, and delivered through optimized administration routes, AAV9 can mediate strong and consistent transgene expression in kidney.

These findings challenge the prevailing assumption that enhanced kidney tropism requires engineered or re-targeted capsids, and instead highlight the importance of holistic vector design, where each component—promoter, cargo, genome configuration, capsid serotype, dose, and delivery route—must be tuned to the physiological and structural features of the target tissue. This work not only expands the toolkit for kidney-directed gene therapy but also provides a practical roadmap for the development of AAV-based therapeutics aimed at treating kidney diseases.

These results underscore the strong therapeutic potential of AAV vectors for kidney-targeted gene delivery. Although LNP-based systems have emerged as prominent non-viral platforms—particularly due to their clinical success in liver-directed applications—their utility for kidney gene transfer remains limited. Multiple studies have reported that both conventional and modified LNPs preferentially accumulate in the liver, especially in hepatocytes, with minimal uptake by the kidney.^23–25^ Despite efforts to enhance kidney tropism through altered lipid compositions, ligand conjugation, and selective organ targeting strategies, LNP-mediated gene delivery to the kidney has generally resulted in low expression levels, poor cell-type specificity, and limited translational potential.^26^ Although the three FDA-approved LNP formulations we assessed herein are known to primarily target the liver via intravenous administration,^27^ the lack of detectable mCherry in kidney using any of our three unbiased quantification methods via both systemic intravenous (Fig. 6) and local intra-arterial administration (data not shown) highlight the current limitations of LNP technologies for kidney targeting. One possible reason for the lack of kidney targeting with LNPs is that LNPs are significantly larger than AAVs (80–100 nanometers vs. 25 nanometers),^28^ which in the absence of acute endothelial injury, likely renders LNPs too large to cross the kidney epithelial-endothelial barrier to gain access to tubular epithelial cells.

Recombinant AAV vectors offer various advantages over LNPs such as prolonged and stable transgene expression following a single administration. Given that the three FDA-approved LNP formulations did not show strong kidney tropism, we sought to evaluate whether the FDA-approved AAV9, which previously has been shown to express only at very low levels in kidney, could show expression through rational capsid engineering and expression cassette design. Among these variables, promoter selection emerged as a major determinant of transgene expression efficiency in the kidney. We compared four seemingly ubiquitous promoters—CBH, CAG, CMV, and EF1α—and found that both CBH and CAG drove markedly stronger expression in proximal tubules compared to CMV or EF1α. This trend was consistent across multiple unbiased quantitative readouts. Although this observation aligns with the findings of Rosales et al., where the CBH promoter drove robust expression in the kidney, in that manuscript, kidney expression was observed only with engineered capsids such as AAV.k13 and AAV.k20, and not with AAV9.^16^ However, Rosales et al. used self-complementary AAV instead of the single-stranded AAV used in Fig. 1 and Fig. 5, and self-complementary AAV is harder to consistently produce with equivalent viral packaging quality.^29,30^ Quantification of viral packaging quality as measured by viral titer or the ratio of full to empty capsids can vary by up to 10-fold between equivalent preparations of self-complementary viruses.^31^ Our quantification data in Fig. 3 show that self-complementary AAV9-Cbh-mCherry expresses with approximately 2x greater intensity than single-stranded AAV9-Cbh-mCherry, but this boost in intensity with self-complementary AAV would be negated if the percentage of full AAV capsids was 10-fold lower in scAAV9-Cbh-mCherry vs. scAAV.k20-Cbh-mCherry. Another factor differing between our studies and those of Rosales et al. is mouse gender. We explicitly used male mice for our experiments whereas Rosales and colleagues used a mix of genders in their studies. Previous work shows that biological sex influences AAV expression in both mice and humans.^32,33^ Due to the estrogen hormone, females tend to mount a more robust immune response to AAV with higher levels of neutralizing antibodies and cytokine/chemokine activity.^32^ Therefore, including more female mice in the scAAV9-Cbh-mCherry group could have caused Rosales et al. to observe a greater difference in mCherry kidney expression between scAAV9-Cbh-mCherry and scAAV.k20-Cbh-mCherry. This sex difference could have been further exacerbated by the fact that Rosales et al. assessed kidney expression with scAAV9-Cbh-mCherry at 30 days post-injection in their mouse studies compared to 21 days in our study, and this longer duration would have given the immune response more time to mount and silence kidney mCherry expression.

Similarly, Furusho et al. reported no expression using the CAG promoter with AAV9 across multiple routes of administration, and required use of the engineered capsid, AAVKP1, to observe tdTomato expression with the CAG promoter in mice.^15^ In contrast, our findings demonstrate that both the CBH and CAG promoters can drive robust and selective expression in mouse kidney even when delivered systemically using AAV9. To account for operator and local mouse strain differences, we validated our mouse results at two institutions- our laboratory in New York and at Stanford University in California (Supplementary Fig. 8). Furusho et al. reported that AAV9-CAG-tdTomato-WPRE expresses in kidney tubular epithelial cells in non-human primates, which is consistent with our tropism data from *ex vivo* normothermic perfusion of live human kidney (Fig. 7). Importantly, significantly larger quantities of AAV9-CAG-tdTomato-WPRE are needed for non-human primate studies than mouse studies since dosing is based on viral genome copies per kilogram body weight. Producing larger AAV quantities often requires using a modified production process and this process typically yields higher quality AAV due to the requirement to remove endotoxins for IACUC approval in non-human primates. Furusho et al. do not address this viral packaging quality issue in their manuscript, which may have contributed to the discrepancy between their AAV9-CAG-tdTomato-WPRE findings across species.

Other investigators have explored the use of kidney cell type–specific promoters to restrict transgene expression to defined nephron segments. Notable examples include the sodium-dependent phosphate transporter type 2a (NPT2a) promoter for proximal tubules, the sodium-potassium-2-chloride cotransporter (NKCC2) promoter for the thick ascending limb of Henle’s loop, and the aquaporin 2 (AQP2) promoter for collecting duct cells.^34^ While these promoters provide high specificity, their relatively large size often limits their compatibility with AAV vectors, particularly in systemically delivered constructs (Supplementary Fig. 5a).

Given the packaging constraints of AAV, our strategy prioritized minimizing the size of regulatory elements without compromising expression efficiency. In this context, we also evaluated the truncated W3 element as a substitute for the full-length woodchuck hepatitis virus post-transcriptional regulatory element (WPRE) (Supplementary Fig. 5b). Importantly, we show that W3 can effectively replace WPRE without loss of expression (Supplementary Fig. 9). This modification increases available space for larger or more complex genetic payloads. Altogether, these results underscore the critical role of expression cassette design in maximizing gene transfer to kidney tubules and support the use of compact configurations such as CBH-W3 when transgene size approaches the AAV packaging limit.

While the design of the expression cassette is essential for enhancing transduction efficiency, our findings underscore that the identity of the cargo may be equally critical for efficacy or quantification of efficacy. Western blot analysis confirmed the presence of GFP protein in kidney tissue, indicating that lack of signal by microscopy does not reflect absent expression but rather limitations in detection *in vivo*—potentially due to reduced brightness, increased autofluorescence, inefficient folding, or altered stability of GFP in the kidney microenvironment. For example, a recent study from Ciobanu et al. showed detectable GFP fluorescence intensity in mouse kidneys that were not injected with AAV1-GFP, highlighting the issue of autofluorescence in kidney.^12^ Ikeda et al. also failed to observe GFP expression in mice treated with AAV8, AAV9, and AAV.Anc80 vectors carrying a CMV-GFP cassette. While GFP expression was detected in the liver and heart, none of these capsids, albeit with the use of a comparatively weak promoter for kidney tubuli, CMV, induced robust fluorescence in the kidney, underscoring the limitations of using GFP as a reporter to evaluate kidney transduction *in vivo.*^35^ Technical improvements to detect GFP fluorescence may well nullify any advantage of mCherry (or other transgene) quantification, but it is reasonable to speculate that interpretation of prior studies of gene delivery optimization using GFP may be confounded by choice of cargo.

Another critical determinant of AAV vector performance is its genome configuration. We directly compared single-stranded (ssAAV) and self-complementary (scAAV) vectors encoding the same expression cassette and observed that, while both formats transduced a similar number of proximal tubules, scAAV9 induced markedly higher fluorescence intensity per tubule. These results suggest that both ssAAV9 and scAAV9 reach a comparable target cell population in the kidney, but scAAV9 achieves substantially greater levels of transgene expression per cell. This finding aligns with the known mechanism of self-complementary AAV vectors, which bypass the rate-limiting step of second-strand DNA synthesis, resulting in faster and more robust transcriptional activation in transduced cells.^36^ Despite this advantage, scAAV vectors are constrained by their reduced packaging capacity (~ 2.4 kb), limiting their application for delivery of larger or more complex genetic constructs. In contrast, ssAAVs support cargo sizes up to ~ 4.7 kb, offering greater flexibility for therapeutic gene delivery.

In an effort to further enhance kidney transduction efficiency, we evaluated a receptor modulation strategy based on neuraminidase pre-treatment. Neuraminidase enzymatically removes terminal sialic acids from the cell surface, thereby unmasking galactose residues known to enhance AAV9 binding in tissues such as skeletal muscle, lung, heart, and liver.^17,37,38^ Although lectin staining confirmed increased exposure of galactose moieties in the kidney following neuraminidase injection, we did not observe a significant increase in the number of mCherry-positive tubules or overall transgene expression. These findings suggest that AAV9 may already have efficient access to its cellular receptors in kidney tubular epithelium under baseline conditions, rendering receptor modulation ineffective in this specific context. We cannot rule out that in states with insufficient exposed galactose residues, neuraminidase pre-treatment may enhance transduction.

To define the optimal serotypes for kidney transduction, we directly compared the performance of four AAV capsids that have recently been reported to drive transgene expression in kidney—AAV9, AAV-KP1, AAV.k20, and AAV.cc47. All four capsids demonstrated similar tropism for the kidney, transducing a comparable number of tubules. To evaluate the impact of viral dose on transduction efficiency, we performed a dose–response analysis using 1×10^10^, 1×10^11^ and 1×10^12^ vector genomes per mouse. We found that the number of transduced tubules plateaued at 1×10^11^ and that increasing the dose to 1×10^12^ resulted in a marked increase in fluorescence intensity within individual tubules, which suggests that higher vector input enhances per-cell or per-tubule expression levels—possibly due to increased vector uptake per cell or improved transcriptional output. These results suggest that, although there may be a biological ceiling for cellular access, intracellular expression levels can be enhanced through dose escalation. Together, these findings emphasize the importance of optimizing both capsid selection and dosing strategy when designing AAV-based therapies for the kidney.

Another critical parameter influencing transduction efficiency is the route of vector administration. Several groups have previously investigated local routes of administration for kidney gene delivery, including intraparenchymal injection,^39^ renal vein injection,^15,40,41^ and retrograde transureteral renal pelvis infusion.^15,42^ Renal artery injection of AAV has been reported by others in rats,^43^ but to our knowledge, our study is the first time that intra-renal artery injection of AAV has been reported in mice. Several groups have successfully employed intra-renal arterial injection in mice to demonstrate efficacy with other non-AAV delivery vehicles.^21,44^ Ullah et al. achieved selective intra-renal delivery of stromal cell–derived vesicles with protective effects in acute kidney injury^21^ and Thin et al. developed a minimally invasive ultrasound-guided approach enabling efficient stem cell delivery and sustained intrarenal retention, but AAV administration via the renal artery is novel to our study.^44^ Surprisingly, we found that systemic intravenous administration yielded higher levels of renal transgene expression than local intra-arterial delivery as measured by unbiased quantification of fluorescence intensity. We used the same AAV dosage for local and systemic delivery; however, because local delivery is administered directly into the kidney, this suggests less efficient transgene expression at the cellular level since the net effective dose going into kidney is significantly higher with local injection. This finding challenges the assumption that local administration necessarily enhances organ-specific targeting and may reflect the rapid clearance of vector through the highly perfused renal circulation or transient hemodynamic changes impairing parenchymal uptake. Similar observations have been reported in other organs, such as the heart, where systemic AAV delivery outperforms local infusion unless combined with pressure-enhancing strategies.^45^ Disruption of the endothelia within the vasa recta, that effectively reduces endothelial cell resistance, as demonstrated in disease states such as acute kidney injury,^46,47^ is an example of a resistance-lowering strategy that may be responsible for observations that intra-arterial kidney delivery may have a higher benefit-to-risk ratio than systemic intravenous delivery of therapeutic payloads to the kidney.^21,48,49^

Interestingly, a study from Furusho et al. tested local kidney injection of AAV9-CAG-tdTomato using two different methods, renal vein injection and retrograde infusion via the ureter, and found that neither method increased tdTomato expression in kidney compared to systemic intravenous injection.^15^ These results corroborate our finding that local administration of AAV9 or AAV.cc47 does not boost expression compared to systemic intravenous injection. However, in the same study when testing the alternative capsid, AAVKP1, the authors found that administering AAVKP1-CAG-tdTomato via retrograde ureter infusion increases kidney expression in proximal tubular epithelial cells by approximately 10-fold. These data from Furusho et al. suggest that the optimal route of administration for achieving kidney gene delivery may be capsid-dependent.

Our data support systemic I.V. injection as the default route for kidney-directed AAV9 delivery, offering the best balance of efficiency, reproducibility, and simplicity under standard conditions. While optimized regional approaches (e.g., renal artery, renal vein, or pelvic retrograde techniques) can achieve kidney transduction in specialized settings, these regional approaches are more invasive, technically variable, and capsid-dependent. In contrast, with an appropriately engineered vector (capsid/cassette) and dose, I.V. administration is sufficient to drive robust proximal-tubule expression without the need for local administration, which requires more specialized expertise to administer than a simple I.V. infusion.

To extend our findings to a clinically relevant setting, we employed a novel, normothermic *ex vivo* perfusion system using discarded human kidneys. In this model, AAV vectors were efficiently sequestered within the kidney parenchyma, with high vector genome copy numbers in tissue and a progressive decrease in viral DNA in the perfusate over time. Notably, this clearance was only partially mirrored by an increase in vector genomes detected in the urine, suggesting that urinary excretion accounts for only a fraction of the viral load. Most vector appeared to be retained within the kidney, as demonstrated by the high vg/dge levels detected in tissue. These findings support a model in which vectors are progressively removed from the circuit, with a portion excreted and a larger proportion taken up by human kidney tissue. These results were further corroborated by detection of DNA by RNAscope^®^ in situ hybridization, which revealed robust transgene tropism in kidney sections exclusively at the end of perfusion, but not at baseline, validating transduction into human kidney epithelia and not confinement to endothelial cells within the kidney vasculature. Importantly, anti-AAV9 antibodies were not detected in perfusate or urine samples at any time point, underscoring the immunological compatibility of the system. Interestingly, there is a complementary line of evidence from a non-human primate autotransplantation study in which AAV9 was delivered *ex vivo* to express the immunomodulator LEA29Y (CTLA4-Ig) and then transplanted back into the non-human primate. In that setting, vector administration achieved mRNA and protein expression at 2 months following re-transplantation with minimal systemic spillover, supporting the feasibility of localized AAV gene delivery to kidneys.^50^ Together, these data establish a robust and human-relevant platform for evaluating AAV-based kidney gene delivery, offering a unique opportunity to assess vector biodistribution, kinetics, and expression prior to clinical translation.

In summary, our work provides a comprehensive framework for the rational design of AAV vectors tailored to kidney-targeted gene delivery. Through systematic evaluation of key parameters—including promoter, cargo, genome configuration, capsid serotype, dose, enzymatic pre-treatment, and route of administration—we define the relative contributions of each component to kidney transduction efficiency. Notably, our findings challenge the prevailing notion that engineered capsids are essential for kidney targeting and instead demonstrate that robust and selective gene expression in tubules can be achieved with conventional AAV9 when combined with optimized expression cassettes, such as CBH-driven constructs incorporating compact regulatory elements like W3.

While further investigation is needed to refine capsid tropism, expand cell-type specificity, and improve vector performance across diverse physiological contexts, the present work establishes a robust experimental foundation for advancing kidney gene therapy. The human *ex vivo* perfusion model introduced here provides a translationally relevant platform for evaluating next-generation vectors directly in human tissue. Our results serve as a practical guide for preclinical vector design and support integration of AAV9-based delivery into emerging gene therapies for treating inherited kidney diseases.

## MATERIALS AND METHODS

### Mouse Experiments

All experimental procedures with live mice were approved by the Institutional Animal Care and Use Committee (IACUC) at either Columbia University (Protocol AC-AABU6656) or Stanford University (Protocol 19287). The aim of our study was to evaluate the expression of mCherry or GFP driven by different promoters using various AAV capsids, delivered through multiple injection routes—including intravenous and intra-renal artery—in mice. Throughout the study, animal care adhered strictly to the ethical standards and regulations set forth by the Columbia University and Stanford University IACUCs. Mice were housed under controlled environmental conditions, with ad libitum access to food and water, a 12-hour light/dark cycle, ambient temperatures maintained between 20–23°C, and relative humidity ranging from 40–50%.

### AAV Vectors and Plasmids

The AAV vectors ssAAV9-CBH (chicken beta actin hybrid)-mCherry-WPRE, ssAAV9-CMV (cytomegalovirus)-mCherry-WPRE, ssAAV9-EF1a (elongation factor 1 alpha)-mCherry-WPRE, ssAAV9-CAG-mCherry-WPRE, and scAAV9-CBH-mCherry-WPRE were obtained from Signagen Laboratories as pre-made AAVs (Catalog #: SL116353, SL101129, SL116405, SL116216, SL116354 and, respectively). ssAAV.cc47-CBH-mCherry-WPRE, ssAAV.cc47-CBH-mCherry-W3, and ssAAV9-CBH-GFP-WPRE were produced by Signagen as custom AAVs using their large-scale production offering, which utilizes a proprietary protocol consisting of triple plasmid transfection in HEK293T cells followed by purification with cesium chloride ultracentrifugation and buffer exchange with dialysis. ssAAVKP1-CBH-mCherry-WPRE and ssAAV.k20-CBH-mCherry-WPRE viruses were produced by Azenta Life Sciences using their 500 μL production scale, which utilizes triple plasmid transfection in a proprietary cell line followed by double purification with iodixanol ultracentrifugation. AAV-KP1 rep/cap plasmid (gift from Mark Kay at Stanford University),^15,18^ AAV.k20 and AAV.cc47 rep/cap plasmids (produced by Genscript), and the AAV-CBH-mCherry-WPRE plasmid (Signagen: SL116353) were used to produce these viruses. The AAV.k20 rep/cap sequence was published by Rosales et al.^16^ The AAV.cc47 rep/cap sequence was published by Gonzales et al.^51^ and has been reported to induce expression in kidney.^20^

### AAV Titration Assay

Viral titers were quantified by real-time PCR (qPCR) using PowerUp^™^ SYBR^™^ Green Master Mix (ThermoFisher, # A257421) and primers specific to the AAV inverted terminal repeats (Forward: GGAACCCCTAGTGATGGAGTT and Reverse: CGGCCTCAGTGAGCGA). Titers were expressed as viral genomes per milliliter (vg/mL).

### AAV vector genome quantification

Total DNA was extracted from perfusate, urine and tissue using the Quick-DNA/RNA Microprep Plus (Zymo Research, #D7005). AAV vector genome copy numbers were quantified by quantitative PCR (qPCR). In brief, 25ng of perfusate, urine and tissue DNA was mixed with Power SYBR Green PCR Master Mix (ThermoFisher, #4367659). We amplified the mCherry and GFP gene sequences using the following primers: mCherry forward (5’-CCACCTACAAGGCCAAGAA-3’), mCherry reverse (5’-CTGTTCCACGATGGTGTAGT-3’), GFP forward (5’-AAGTTCATCTGCACCACCG-3’), GFP reverse (5’- TGCTTGTCGGCCATGATATAG-3’) Actb forward (5’- CATTGCTGACAGGATGCAGAAGG-3’) and Actb reverse (5’- TGCTGGAAGGTGGACAGTGAGG-3’). Information on copy number standards and normalization for mouse study was described previously.^15^ Vector genomes copy numbers were expressed as double-stranded vector genome copy numbers per diploid genomic equivalent (vg/dge).

### Intravenous administration in wild-type C57BL/6J mice

For all mouse studies, 10-week-old male and female adult mice were systemically administered with AAV or Lipid Nanoparticles (LNPs) via the tail vein, as indicated in the text. LNPs formulated with mCherry-encoding mRNA, ALC-0315, SM-102 and DLin-MC3, were produced by MilliporeSigma and injected at a dose of 25 μg per mouse (~ 1 mg LNP/kg body weight). For AAV injections, doses ranged from 1.0 × 10^10^ to 1.0 × 10^12^ vg/mouse, depending on the experimental group (1.0 × 10^10^ vg, 1.0 × 10^11^ vg, 2.0 × 10^11^ vg, or 1.0 × 10^12^ vg per mouse). For neuraminidase pre-treatment experiments, mice received an intravenous injection of 200 milliunits of neuraminidase (MilliporeSigma) two hours prior to AAV administration. Mice were euthanized at the time point indicated, and the kidneys, liver, spleen and heart were collected for histological analysis via immunofluorescence microscopy.

### Intra-renal artery administration in wild-type C57BL/6J mice

For intra-renal artery delivery studies, male and female C57BL/6J mice were anesthetized with isoflurane and placed on a warming pad. Under sterile conditions, a midline laparotomy was performed, and the abdominal aorta was exposed. For all surgeries, 100 μL of tattoo dye, consisting of a 1:1 ratio of Solvent Green 3 (95% dye content) and 1 mg/mL polymethine dye (Sigma-Aldrich, USA), was used as a surrogate marker to assess the distribution of the therapeutic solution. As described in Ullah et al. (Cells, 2020), three temporarily **6** – 0 silk sutures were positioned around the proximal aorta, distal aorta, and superior mesenteric artery (SMA) to isolate renal circulation. Once ready for injection, sutures were briefly tightened in sequence to limit blood flow to the kidneys for under 3 minutes. A 36-gauge needle was inserted into the aorta just distal to the left renal artery, and a total of 100 μL of solution (50 μL per kidney) was slowly injected. LNPs formulated with mCherry-encoding mRNA, ALC-0315, SM-102 and DLin-MC3, were produced by Merck KGaA, Darmstadt, Germany, and injected at a dose of 25 μg per mouse (~ 1 mg LNP/kg body weight). For AAV injections, a dose of 1.0 × 10^11^ vg/mouse was used for all intra-renal artery injections. For neuraminidase pre-treatment experiments, mice received an intravenous injection of 200 milliunits of neuraminidase (MilliporeSigma) two hours prior to AAV administration. A transient microvascular clamp was applied to the left renal artery during the injection to promote distribution to the right kidney. Control mice underwent the same surgical procedure with PBS only. Following injection, hemostasis was achieved with gentle pressure, sutures were released in reverse order (distal aorta, SMA, proximal aorta), and the abdomen was closed with **5** – 0 absolvable sutures and wound clips. Mice received preoperative buprenorphine SR for analgesia and were monitored on supplemental heat until fully recovered. To accommodate potential reduced mobility post-surgery, gel food was placed on the cage floor. Mice were euthanized at the time point indicated, and the kidneys, liver, spleen and heart were collected for histological analysis via fluorescence microscopy.

### Histological Processing and Analysis

Harvested organs were fixed overnight in 10% neutral-buffered formalin (X), followed by cryoprotection in either 30% sucrose (X) or storage in 70% ethanol. Tissues cryoprotected with sucrose were embedded in FSC 22 Frozen Section Media (Leica #3801480), sectioned at 6 μm thickness using a cryostat. Sections were analyzed by immunofluorescence and light microscopy.

For immunostaining, tissue sections were outlined using a Liquid Blocker Super PAP Pen and incubated with Carbo-Free^™^ Blocking Solution (5X) supplemented with 0.3% Triton X-100 (Sigma, #T8787) for 1 h at room temperature. Sections were washed three times in PBS (ThermoFisher, 20012–027) during 5 minutes and incubated overnight at 4°C with: LTL-fluorescein (1:100; Vector Laboratories, FL-1321–2), biotinylated Dolichos biflorus agglutinin (DBA, 1:250; Vector Laboratories, B-1035–5), Erythrina Cristagalli Lectin (ECL,ECA) Fluorescein (FL-1141–5) (1:250; Vector Laboratories, FL-1141–5) and/or Maackia Amurensis Lectin I (MAL I), Biotinylated (B-1215–2) (1:250; Vector Laboratories, B-1215–2) as indicated. After incubation, sections were washed (3 × 5 min in PBS). Sections were further incubated for 1 h at room temperature with Streptavidin-Cy5 (1:100; Vector Laboratories, SA-1500–1-1 mg). After three additional PBS washes, sections were incubated with DAPI (ThermoFisher, D3571) for 5 min at room temperature, followed by final PBS washes (3 × 5 min). Sections were mounted with Diamond Antifade Mountant (ThermoFisher, P36965), coverslipped, and cured for 24 h at room temperature.

Immunofluorescence images were acquired using either an EVOS M5000 Imaging System or a Nikon AXR MP/Ti2. Quantification of mCherry-positive tubules was performed using a novel machine learning-guided image analysis tool developed in the Roboflow platform, with a minimum of ten images analyzed per kidney. Quantification of mCherry-intensity tubules was performed using ImageJ, with a minimum of ten images analyzed per kidney.

### Lipid Nanoparticle Formulation

Three different lipid nanoparticle (LNP) formulations were produced by MilliporeSigma (Merck KGaA, Darmstadt, Germany) under a small-scale manufacturing service agreement. Each formulation employed a unique ionizable lipid combined with standard helper lipids — DSPC (1,2-distearoyl-sn-glycero-3-phosphocholine; Avanti Polar Lipids), cholesterol (Sigma-Aldrich), and PEGylated lipid (PEG2000-DMG or ALC-0159; Avanti Polar Lipids) — following the compositions of three market-approved LNP platforms (Comirnaty^®^, Spikevax^®^, and Onpattro^®^).

Formulations were as follows: SM-102-based LNPs: SM-102 (9-Heptadecanyl 8-{(2-hydroxyethyl)[6-oxo-6-(undecyloxy)hexyl]amino}octanoate) as ionizable lipid, DSPC as phospholipid, cholesterol as helper lipid, and PEG2000-DMG as PEGylated lipid, combined at a 50:10:38.5:1.5 mol% ratio. ALC-0315-based LNPs: ALC-0315 ((4-hydroxybutyl)azanediyl)bis(hexane-6,1-diyl)bis(2-hexyldecanoate)) as ionizable lipid, DSPC as phospholipid, cholesterol as helper lipid, and ALC-0159 as PEGylated lipid, at a 47.5:10:40.7:1.8 mol% ratio. DLin-MC3-DMA-based LNPs: DLin-MC3-DMA (4-(dimethylamino)butanoic acid, (10Z,13Z)-1-(9Z,12Z)-9,12-octadecadien-1-yl-10,13-nonadecadien-1-yl ester) as ionizable lipid, DSPC as phospholipid, cholesterol as helper lipid, and PEG2000-DMG as PEGylated lipid, at a 50:10:38.5:1.5 mol% ratio.

Upon completion, formulations were characterized for particle size, polydispersity index (PDI), mRNA encapsulation efficiency, and stability following three freeze–thaw cycles and storage at − 80°C. Each LNP batch was supplied at a final concentration of 1 mg mCherry mRNA/mL and used within one week after thawing. mCherry mRNA was provided by Genscript. For *in vitro* experiments, we administered 5 μg per well in a 6-well plate that was seeded at 30,000 cells/well approximately 24 hours later. We imaged the cells for mCherry expression at day 24 and day 72. For *in vivo* experiments, we dosed ten-week-old mice with 25 μg of LNP mRNA per mouse (~ 1 mg/kg dosage) via tail vein injection.

### Immunoblot analysis for GFP expression in mice

Mouse kidneys from ssAAV9-CBH-mCherry-WPRE, ssAAV9-CBH-GFP-WPRE and ssAAV9-CBH-mCherry + WPRE, ssAAV9-CBH-GFP-WPRE treated mice and were lysed by adding 500 μl of T-PER^™^ Tissue Protein Extraction Reagent Pierce Protease (ThermoFisher, # 78510) and Phosphatase Inhibitor Mini Tablets (ThermoFisher, # A32959). Add one Mini Tablets per 7ml of TPER before mechanical lysis using a VWR – 10158–558-EACH homogenizer (Neobits). Lysate was cleared by centrifugation (16,000 × g, 2 min, 4°C) to remove remaining debris. Samples were incubated at 95°C for 5 min in LDS loading buffer supplemented with 10% β-mercaptoethanol, separated by NuPAGE^™^ Bis-Tris Mini Protein Gels, 10%, 1.0–1.5 mm (ThermoFisher, #NP0301BOX) and transferred with Invitrogen^™^ iBlot^™^ 2 Gel Transfer Device (ThermoFisher, #IB21001) using iBlot^™^ 2 Transfer Stacks, nitrocellulose, mini (ThermoFisher, #IB23002) program P0. Membranes were blocked with 5% milk powder in Tris buffered saline with − 0.1% Tween 20 (TBST) and incubated for 1 h at room temperature. Membranes were subsequently incubated with primary antibodies for GFP (1:1,000; ThermoFisher, #A-11122) or Anti-β-Actin (1:50,000; Millipore Sigma, #A3854) in 2.5% milk in TBST overnight at 4°C. The following day, membranes were washed 3× for 5 min with TBST. Membranes were then incubated with goat anti-rabbit IgG (1:1,000; Jackson ImmunoResearch, 111–035-144) in 2.5% milk in TBST for 1 h at room temperature. Membranes were washed 3× for 5 min with TBST and incubated for 1 min with enhanced chemiluminescence substrate (ThermoFisher, #34580). Immunoblots were visualized on a iBright FL1000 Imaging Systems.

### Normothermic Ex Vivo Kidney Perfusion

Normothermic *ex vivo* perfusion was conducted by Revalia Bio (New Haven, CT, USA) in line with standard clinical kidney perfusion protocols as previously described.^52^ Deceased donor kidneys were used, so these experiments are explicitly not human subjects data and are therefore IRB exempt. We used a kidney from a male deceased donor with a weight of 259 grams.

### RNAscope^®^ Assay on FFPE human kidney tissue

Formalin-fixed, paraffin-embedded (FFPE) tissue sections were prepared from human kidney samples collected at the start and end of the *ex vivo* perfusion experiment. RNAscope^®^ in situ hybridization was performed using the RNAscope^®^ 2.5 HD Detection Reagent – RED Kit (Advanced Cell Diagnostics, ACD Bio) following the manufacturer’s protocols for FFPE sample preparation and pretreatment (PART 1, Document No. 322452) and hybridization/detection steps (PART 2, Document No. 322360-USM).

The following probes were used for signal detection: Negative control: DapB (Cat. No. 310043) and positive control: PPIB (Cat. No. 313901). Experimental probes: mCherry-sense-C1 (Cat. No. 1838221-C1) and EGFP-O4-sense-C1 (Cat. No. 1040801-C1). Importantly, sense probes were used to detect DNA instead of RNA that are typically detected by anti-sense probes. Images were acquired using Leica Aperio AT2 and ThermoFisher EVOS S1000 microscopes, and signal detection was visualized as red dots indicating probe-specific transcripts. Blue counterstaining was used to label nuclei.

### Blinding

The following procedures were conducted in a blinded fashion in which the investigator remained unaware of the specifics of the vectors and samples: AAV injections and fluorescence microscopy. Although other experiments were conducted in an unblinded fashion, efforts were made to minimize bias and ensure objective observations.

### Statistical analysis

GraphPad Prism software (v.10.4.2) was used for statistical analysis. All values are presented as mean ± s.e.m. For data sets with two groups, significance was determined using unpaired t test. For data sets with more than two, significance was determined using one-way analysis of variance (ANOVA). *P* < 0.05 was considered statistically significant. Only statistically significant *P* values are reported.

### Machine-learning-based image analysis tool

Whole-section kidney images (mCherry channel overlaid on bright-field) were quantified with an instance segmentation pipeline in Roboflow. Tubules were manually annotated with free-hand polygons on the bright-field outline; glomeruli, vessels, edge artifacts, and ambiguous structures were excluded. Two mutually exclusive classes were defined: 1) mCherry-positive: tubules showing a continuous mCherry signal along ≥ 50% of the epithelial perimeter or a clear above-background intensity in ≥ 50% of the epithelial/luminal area. 2) mCherry-negative: tubules with absent or sub-threshold signal (< 50%). The curated dataset contained 223 total images with 154 images assigned to the training set, 47 images assigned to the validation set, and 22 images assigned to the test set. Model was trained in Roboflow using Roboflow 3.0 – Instance Segmentation (Accurate). Evaluation metrics were mAP@50 = 78.3%, precision = 69.7%, and recall = 77.0% (Roboflow Models panel). For inference, thresholds were fixed across all experiments: confidence = 0.50, IoU/overlap = 0.50, opacity = 0.75 (Roboflow Preview Model). An illustrative annotation example is provided in Supplementary Fig. 1a-b.

## Supplementary Material

Supplementary Files

This is a list of supplementary files associated with this preprint. Click to download.

• SupplementaryV2.docx
